# An Analysis of Real, Self-Perceived, and Desired BMI: Is There a Need for Regular Screening to Correct Misperceptions and Motivate Weight Reduction?

**DOI:** 10.3389/fpubh.2017.00012

**Published:** 2017-02-08

**Authors:** Jonathan F. Easton, Christopher R. Stephens, Heriberto Román Sicilia

**Affiliations:** ^1^C3 – Centro de Ciencias de la Complejidad, Universidad Nacional Autónoma de México, Ciudad de México, México; ^2^Instituto de Ciencias Nucleares, Universidad Nacional Autónoma de México, Ciudad de México, México

**Keywords:** self-perception, BMI, figure rating scale, perceived weight, desired weight, diagnosis of obesity

## Abstract

We study the relationship among real, self-perceived, and desired body mass index (BMI) in 21,288 adults from the Mexican National Health and Nutrition Survey 2012, analyzing the effect of sex and diagnosis of obesity/overweight by a healthcare professional. Self-perceived and desired BMI are analyzed via a figure rating scale question and compared to real BMI. Only 8.8 and 6.1% of the diagnosed and non-diagnosed obese, respectively, correctly identify themselves as such. For the obese, 20.2% of non-diagnosed and 12.7% of diagnosed perceive themselves as normal or underweight, while 49.1 and 37% of these are satisfied with their perceived BMI. Only 7.8% of the obese, whose real and perceived BMI coincide, have a desired BMI equal to their perceived one. In contrast, 43.2% of the obese, whose perceived BMI is normal, have a desired BMI the same as their perceived one. Although the average desired body figure corresponds to the normal BMI range, misperceptions of BMI correlate strongly with the degree of satisfaction associated with perceived BMI, with larger misperceptions indicating a higher degree of satisfaction. Hypothesizing that the differences between real, perceived, and desired weight are a motivator for weight change, one potential intervention could be the periodic assessment of real, perceived, and desired BMI in order to correct misleading weight misperceptions that could potentially obstruct positive behavioral change.

## Introduction

Obesity is one of the most complex health problems worldwide, being a fast growing problem (1) that affects both adults and children alike (2, 3). An important reason why obesity is such a hard problem to tackle is its multifactorial nature. There exist multiple risk factors at all scales: from the microscopic, such as genetic factors (4), to macroscopic factors, such as nutrition and exercise (5, 6). Additionally, as obesity is a slowly evolving condition, where symptoms, such as weight gain, tend to occur in small increments over a period of many years, it can be difficult for people to notice significant weight gain until they are already overweight or obese. It is therefore potentially important that people are made aware of significant weight gain so that preventative interventions can be made in an attempt to stop, or at least slow, the trend before obesity is reached.

One important group of potentially causal factors associated with weight gain and, following that, obesity, is the self-perception of weight and weight gain and its relation to real weight and desired weight. The relation between self-perception of weight, desired weight, and real weight was the focus of this investigation. Of course, it is important for a population to acknowledge that obesity is a serious condition with important health consequences and, more specifically, that weight gain is a serious symptom as opposed to a passing phase or an inevitability of aging (7). However, it is equally important that people can accurately perceive, evaluate, and contextualize their real weight, and any significant weight gain, so as to be able to act appropriately before the obese state is reached. For instance, if a person’s perceived weight is also their desired weight, but is far less than their real weight, one might surmise that there would be less motivation for weight change. Thus, we hypothesize that adverse discrepancies between real, i.e., measured, self-perceived, and desired body mass index (BMI) may play a significant role in the obesity epidemic by obfuscating an individual’s true health state and therefore inhibiting appropriate and necessary behavioral and lifestyle changes. Self-perception itself is, of course, complex, involving not only how a person sees him/herself but also how their view of normality is affected by the state of their social or family group and even up to the general population. For example, if a person is very overweight, but all of their friends and colleagues are overweight too, their opinion of themselves may be that they are of an acceptable and normal weight, due to the state of the population around them (8).

A number of techniques exist for the analysis of self-perception of BMI. The most commonly used format is a figure rating scale (FRS) [see Ref. (9) for a review], which offers a good technique for self-assessment of body figure which, in turn, can be approximately related to a corresponding BMI. Another type of self-perception question, based on direct weight category (10), is also common. There is a great deal of variation in the specific FRSs used, from those using 10 pictorial (11) or photographic images (12) to scales for specific races/sexes (13). A much used FRS is the Stunkard scale (14), an FRS of nine figures split for sex. This FRS has been argued to be a very good scale for the analysis of self-perception of weight and BMI (15), while FRSs in general have been taken to be reliable proxies for body figure, BMI, and weight (16).

Studies of self-perception of weight and body figure are very wide ranging, taking in factors such as weight control and income for the British (17), or focusing on the use of an FRS for Mexican men and women (18). The extension to comparisons between real, self-perceived, and desired weight is more uncommon, especially in the context of large populations, hence the motivation for this paper. However, some studies with small sample sizes do exist (19, 20). In the case of Mueller et al. (19), the comparison between real and desired weight is made for a sample of 257 women and 251 men, based on four weight categories, from underweight to obese, where both desired weight and real weight is self-reported. The conclusion drawn is that desired weight loss increased with real BMI. Examples of studies can be found using an FRS, such as in Neighbors and Sobal (20), who considered a sample of 310 adults. There the analysis focused more directly on body shape and body weight dissatisfaction for both sexes. Results showed that females have greater body dissatisfaction than men, while in terms of real BMI the overweight showed the most dissatisfaction. The main conclusion drawn is that sociocultural factors affect how men and women view themselves in terms of body weight, with women preferring small body sizes, while the men tend to prefer slightly larger body sizes, preferring a muscular physique.

In this paper, we explored the relations among three BMI/body figure variables—real BMI, perceived body figure, and desired body figure—with the benefit of a large dataset of 21,288 adults, taken from the Mexican National Health and Nutrition Survey (ENSANUT) 2012 (21). Our first aim was to study self-perception of body figure compared to real BMI and how a previous diagnosis of obesity and/or sex affects self-perception. Our second aim was to extend this analysis to also compare perceived and desired body figures on an FRS for these adults.

## Materials and Methods

### Data

The Mexican ENSANUT 2012 survey was used for this analysis. ENSANUT 2012 is the most recent nationwide survey of Mexico, containing data from all states and covering a range of topics including: nutrition, sociodemographics, medical histories, home life, and many more (21). The survey was conducted such that the data provide an adequate and varied representation of the Mexican population. The ENSANUT 2012 survey data were preprocessed by starting with all individuals who have responses to the health and nutrition survey, exclusions were first made based on missing data for any of the required variables for this study, in the following order: missing data for height or weight (*n* = 9,695), FRS responses for perceived or desired state (*n* = 831), and responses to the medical diagnosis questions (*n* = 141). Adults with values of BMI outside the range 13.5–60 kg/m^2^ (*n* = 34) were excluded due to their highly atypical nature. Finally, those remaining individuals who had been diagnosed as diabetic (*n* = 2,219) were also removed, due to the comorbidity of obesity and diabetes, in order to make sure the diagnosis of diabetes had no effect on the results of this study. No exclusions were made based on age where the age range was from 20 to 101 for both men and women with an average of 43.3 and 41.5, respectively. The final data used 21,288 adults, comprising 59.71% women to 40.29% men, reflecting the proportions of the full ENSANUT 2012 dataset.

The data were split into four groups, based on sex and on a previous diagnosis of obesity/overweight by a healthcare professional. Diagnosis by a medical professional was self-reported and consisted of those adults who had been tested in the last 12 months for presence of obesity/overweightness and for whom the outcome of the test was positive, i.e., obese/overweight. No further information was available as to why a given individual received a screening. Due to a lack of information, we are unable to test accurately whether the diagnosis of obesity was a single diagnosis or a by-product of a diagnosis of another health condition, such as diabetes. We assume that other health conditions do not affect self-perceived and desired body weight. The non-diagnosed were defined as those who had not had a test, together with those who had taken a test but received a negative diagnosis. In secondary analysis, we checked that excluding the participants with a normal BMI at a previous screening from the category of non-diagnosed participants did not significantly change the interpretation of the results. Real BMI does not factor into the categorization of the diagnosed as it is the effect of a diagnosis by a medical professional that is relevant in this case. The four groups that we considered were: non-diagnosed men (*n* = 7,756), diagnosed men (*n* = 820), non-diagnosed women (*n* = 10,968), and diagnosed women (*n* = 1,744). For each of these groups, the individuals may or may not have a real BMI corresponding to the obese or overweight categories. Thus, an individual may have been diagnosed overweight/obese in the last 12 months but since then reduced their BMI to a normal level. A breakdown of the data can be seen in Table 1, where each of the four main categories are split into three current BMI states: normal (BMI < 25 kg/m^2^), overweight (25 ≤ BMI < 30 kg/m^2^), and obese (BMI ≥ 30 kg/m^2^).

**Table 1:** **Summary sample sizes for the data used from the ENSANUT 2012 survey (*n* = 21,288)**.

ND men (7,756)			D men (820)			ND women (10,968)			D women (1,744)		
Normal	Over	Obese	Normal	Over	Obese	Normal	Over	Obese	Normal	Over	Obese
2,601	3,292	1,863	27	281	512	3,205	4,130	3,633	66	487	1,191
33.54%	42.44%	24.02%	3.29%	34.27%	62.44%	29.22%	37.65%	33.12%	3.78%	27.92%	68.29%

*ND, non-diagnosed; D, diagnosed by a medical professional. Normal/over/obese for real BMI*.

### Figure Rating Scale and BMI

**Figure 1:**
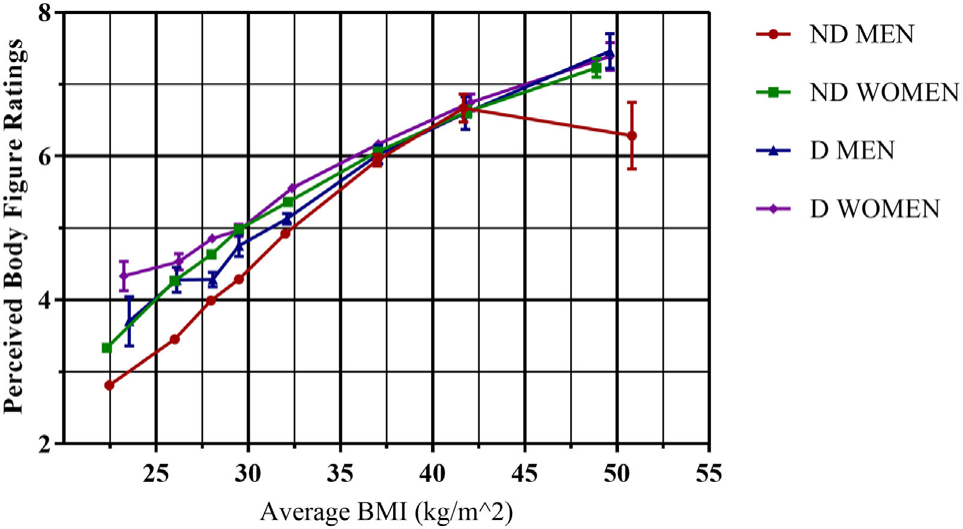
**Comparison of non-diagnosed versus diagnosed obese mean responses for the Stunkard scale perceived body figure rating question, by sex**. Standard error bars are included for each point for body figure response. ND, non-diagnosed; D, diagnosed. Figures 1 and 2, underweight; figures 3 and 4, normal; figures 5–7, overweight; figures 8 and 9, obese.

**Figure 2:**
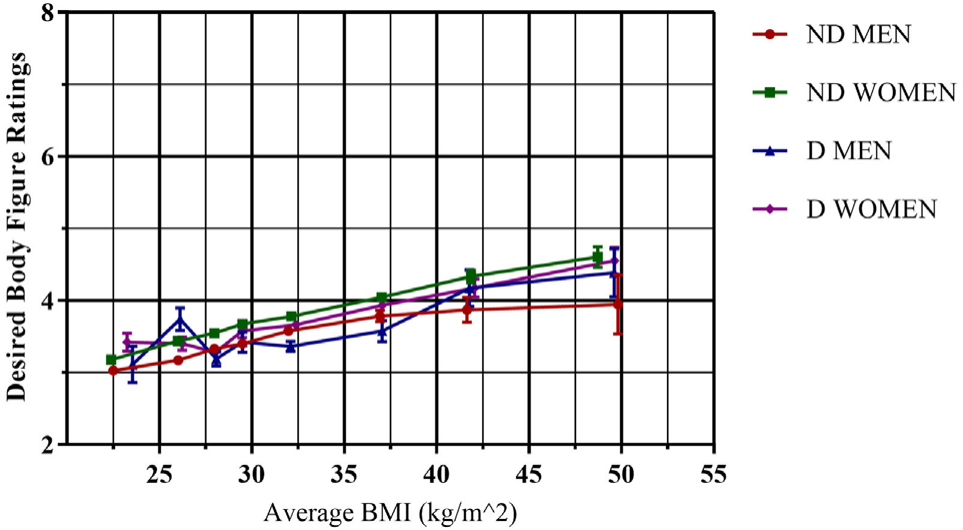
**Comparison of non-diagnosed versus diagnosed obese mean responses for the Stunkard scale desired body figure rating question, by sex**. Standard error bars are included for each point for body figure response. ND, non-diagnosed; D, diagnosed. Figures 1 and 2, underweight; figures 3 and 4, normal; figures 5–7, overweight; figures 8 and 9, obese.

The method of testing self-perception of BMI in the ENSANUT 2012 dataset is through an FRS question. The specific FRS used is a version of the nine figure Stunkard scale (14). The question itself has two parts, based on separate scales for men and women. The first part of the question asks the participant to select which body figure they feel most represents them at this moment. The second part of the question asks the respondent to select the body figure which they would prefer to be. The nine body figures in the scale can be split into four main categories by BMI. Figures 1 and 2 represent underweight, figures 3 and 4 are normal, figures 5–7 are classed as overweight, and figures 8 and 9 as obese (22, 23). In this way, if we assume a linear relationship between FRS response and weight classification, we can use the FRS as a proxy for perceived or desired BMI for each individual and therefore make a comparison with real BMI. Thus, if a person’s perceived BMI was a faithful representation of their real BMI, we should observe that passing from BMI normal to BMI obese corresponds to a change in average FRS response from 3–4 to 8–9.

A trained interviewer measured height (meter) and weight (kilogram) during the questionnaire and from this, BMI (kilogram/meter^2^) was calculated using the standard formula of weight divided by the square of the height. BMI itself was divided into eight discrete groups: 13 ≤ BMI < 25, 25 ≤ BMI < 27, 27 ≤ BMI < 29, 29 ≤ BMI < 30, 30 ≤ BMI < 35, 35 ≤ BMI < 40, 40 ≤ BMI < 45, and 45 ≤ BMI ≤ 60. Eight groups were chosen in order to give both an ample range of values for comparison and an adequate sample size in each group to preserve statistical power. When creating the ranges of the eight discrete BMI groups, the standard boundaries for BMI categories were adhered to for ease of analysis of the results, i.e., BMI = 25 kg/m^2^, the boundary between normal and overweight, and BMI = 30 kg/m^2^, the boundary between overweight and obesity stage 1.

### Hypothesis and Approach

The null hypothesis, for both men and women, was that there exists no difference between the mean self-perception responses of a non-diagnosed obese/overweight and a diagnosed obese/overweight Mexican adult, for each BMI range. Therefore, there were two main comparisons to be made: to the difference between non-diagnosed and diagnosed; and for both men and women. For completeness, we also tested the same hypothesis in relation to the desired body figure on the FRS. To study the effect of a previous diagnosis of overweight/obesity by a medical professional on self-perception, the average body figure response for each BMI range was calculated for each of the four groups: non-diagnosed men, non-diagnosed women, diagnosed men, and diagnosed women. The average response was plotted versus average real BMI within the eight BMI groups. The same analysis was also carried out for the desired body figure.

Following this, a direct comparison of self-perceived body figure and desired body figure was made for each individual, in order to determine whether a diagnosis of overweight/obesity, or sex, affects body image satisfaction. We classified individuals into: those whose perceived body figure rating is less than their desired body figure rating; equal to their desired body figure rating; or greater than their desired body figure rating. We calculated the percentage of adults in each of these categories for each diagnostic category—diagnosed obese/overweight and non-diagnosed and for each BMI class—obese (BMI ≥ 30 kg/m^2^), overweight (25 ≤ BMI < 30 kg/m^2^), and normal (BMI < 25 kg/m^2^). Thus, we may simultaneously observe the relation between real BMI, perceived BMI, and desired BMI. Percentages were calculated across each body figure and for each BMI category so as to total 100%. The use of only three BMI categories in this analysis was to increase statistical power by having more individuals in the breakdown of data across all nine body figures with a previous diagnosis.

All differences were checked for statistical significance, where the corresponding *p*-values showing the level of significance are given for each test. An independent *T*-test was used when comparing the mean responses across the BMI ranges for sex and diagnosis. Second, a chi-squared test was used when comparing percentages, as was necessary for the results of the perceived versus desired analysis.

## Results

### Perceived Body Figure Rating

The frequency of obesity given by real BMI was 24.02 and 33.12% for non-diagnosed men and women, respectively, while the proportions of all men and women that have been diagnosed as obese or overweight were 9.56 and 13.72%. The proportions of men/women who after an obese/overweight diagnosis are associated with a normal real BMI were 3.29 and 3.78%, respectively. Assuming that following diagnosis of obesity/overweight by a medical professional the newly diagnosed patient was instructed to lose weight, these percentages are very low. Although the obese/overweight diagnosis was in the last 12 months, and so any weight loss attempt would have been limited in time, this still supports the reported fact that in practice, and even without time constraints, it is very difficult to lose weight and maintain it (24).

In Figure 1, we see that the average body figure response, considered as a proxy for perceived BMI, was a monotonically increasing, non-linear function of real BMI. It is evident that in the region of approximately 20 ≤ BMI < 35 kg/m^2^, there is a strong linear tendency, after which there was a slight drop in inclination for all four categories, particularly in the case of the non-diagnosed men. We also see a significant offset between the women’s and men’s curves in that for a given real BMI men appeared to have a generally lower self-perception on the FRS than the women, though this difference decreases with increasing BMI. Each of the first five BMI ranges showed a significant difference (*p* < 0.001) in an independent *T*-test comparing men and women for the diagnosed and the non-diagnosed separately. This was particularly of note for the BMI < 25 kg/m^2^ group, with women across both diagnostic groups identifying themselves on average as 0.5 body figure rating units higher than men, signifying a difference in what body figure rating is considered “normal.” The corresponding difference between diagnosed and non-diagnosed was slightly higher. It is notable that these biases between different groups diminish as a function of real BMI, with the differences becoming statistically insignificant for ranges of BMI ≥ 35 kg/m^2^, indicating that the self-perception of people with morbid obesity is independent of diagnosis or sex. The final three real BMI ranges have *p* = 0.02, *p* = 0.753, and *p* = 0.814, respectively, when comparing sexes with non-diagnosed and diagnosed considered together. Non-diagnosed men were not included in the analysis for the final BMI range due to their uncharacteristic drop in average body figure response.

### Desired Body Figure Rating

Figure 2, by contrast, shows the corresponding analysis for desired body figure. Notable was the fact that desired body figure showed relatively little change as real BMI increases, indicating an almost universal average desired body shape for both men and women that is exclusively in the normal range, figure rating 3 and 4, for real BMI < 35 kg/m^2^. It is interesting to note however that there is a definite but weak increase in desired BMI as a function of real BMI. Desired BMI for women can be seen to be approximately 0.3 figure rating units higher than men over the range 20 ≤ BMI < 35 kg/m^2^. There is no systematic significant difference between the diagnosed and non-diagnosed across the full BMI range. The results of an independent *T*-test show that there was only a significant difference between the diagnosed and non-diagnosed for males in the second BMI range (*p* = 0.001), which is reflected in Figure 2. The case for non-diagnosed versus diagnosed women is similar, with no significant difference in desired body on a range-by-range basis, apart from the ranges 27 ≤ BMI < 29 kg/m^2^ (*p* = 0.001) and 30 ≤ BMI < 35 kg/m^2^ (*p* = 0.031).

### Perceived versus Desired Body Figure Rating

Table 2 shows the results of the perceived versus desired BMI analysis. The results confirmed the known result that the obese/overweight generally underestimate their BMI (19, 25), with only 6.1% of the non-diagnosed obese correctly identifying themselves in body figure categories 8 and 9 for obesity and 41% in categories 5, 6, and 7 for overweight. The corresponding percentages for the diagnosed were 8.8 and 55.4%. Interestingly, however, the percentage of participants with BMI < 25 kg/m^2^ that identified themselves correctly in body figure categories 1–4 is 88.5% for the non-diagnosed and only 59.1% for the diagnosed. This also confirms the results seen in Figure 1.

**Table 2:** **Comparison of perceived versus desired body figure rating, measured on the Stunkard scale**.

Non-diagnosed																		
	BMI ≥ 30 kg/m^2^ (***n*** = 5,496)						25 ≤ BMI < 30 kg/m^2^ (***n*** = 7,422)						BMI < 25 kg/m^2^ (***n*** = 5,806)					
	*P* > Des		*P* = Des		*P* < Des		*P* > Des		*P* = Des		*P* < Des		*P* > Des		*P* = Des		*P* < Des	
Fig	*n*	%	*n*	%	*n*	%	*n*	%	*n*	%	*n*	%	*n*	%	*n*	%	*n*	%
1	0	0.00	21	58.33	15	41.67	0	0.00	216	66.06	111	33.94	0	0.00	427	54.53	356	45.47
2	30	28.30	63	59.43	13	12.26	76	13.64	379	68.04	102	18.31	46	3.94	711	60.87	411	35.19
3	81	38.39	109	51.66	21	9.95	264	24.72	697	65.26	107	10.02	199	12.78	1,057	67.89	301	19.33
4	454	59.97	287	37.91	16	2.11	1,186	49.94	1,128	47.49	61	2.57	581	35.58	915	56.03	137	8.39
5	1,357	82.79	272	16.60	10	0.61	1,520	74.88	495	24.38	15	0.74	289	58.03	194	38.96	15	3.01
6	1,472	89.54	165	10.04	7	0.43	700	79.91	169	19.29	7	0.80	95	67.38	42	29.79	4	2.84
7	714	92.97	53	6.90	1	0.13	124	88.57	14	10.00	2	1.43	12	80.00	3	20.00	0	0.00
8	218	92.77	16	6.81	1	0.43	25	92.59	2	7.41	0	0.00	5	83.33	1	16.67	0	0.00
9	90	90.00	10	10.00	0	0.00	18	81.82	4	18.18	0	0.00	5	100.00	0	0.00	0	0.00

**Diagnosed**																		
	**BMI ≥ 30 kg/m^2^ (***n*** = 1,703)**						**25 ≤ BMI < 30 kg/m^2^ (***n*** = 768)**						**BMI < 25 kg/m^2^ (***n*** = 93)**					
	***P* > Des**		***P* = Des**		***P* < Des**		***P* > Des**		***P* = Des**		***P* < Des**		***P* > Des**		***P* = Des**		***P* < Des**	
**Fig**	***n***	**%**	***n***	**%**	***n***	**%**	***n***	**%**	***n***	**%**	***n***	**%**	***n***	**%**	***n***	**%**	***n***	**%**
1	0	0.00	7	77.78	2	22.22	0	0.00	3	50.00	3	50.00	0	0.00	4	57.14	3	42.86
2	3	15.00	13	65.00	4	20.00	4	20.00	13	65.00	3	15.00	0	0.00	8	80.00	2	20.00
3	21	60.00	13	37.14	1	2.86	29	42.03	32	46.38	8	11.59	3	23.08	9	69.23	1	7.69
4	112	73.68	38	25.00	2	1.32	167	70.46	68	28.69	2	0.84	14	56.00	11	44.00	0	0.00
5	420	92.72	33	7.28	0	0.00	245	90.07	25	9.19	2	0.74	15	83.33	3	16.67	0	0.00
6	565	95.93	23	3.90	1	0.17	129	96.99	4	3.01	0	0.00	12	92.31	1	7.69	0	0.00
7	286	96.95	8	2.71	1	0.34	19	90.48	2	9.52	0	0.00	5	100.00	0	0.00	0	0.00
8	105	98.13	2	1.87	0	0.00	7	87.50	1	12.50	0	0.00	1	100.00	0	0.00	0	0.00
9	43	100.00	0	0.00	0	0.00	2	100.00	0	0.00	0	0.00	1	100.00	0	0.00	0	0.00

*BMI, body mass index; P, perceived figure rating; Des, desired figure rating; Fig, figure rating. Figures 1 and 2, underweight; figures 3 and 4, normal; figures 5–7, overweight; figures 8 and 9, obese*.

*Percentages are calculated for each body figure dependent on whether the perceived rating is higher (*P* > Des), equal to (*P* = Des), or lower (*P* < Des) than the desired rating*.

There are two main patterns that exist in Table 2. For participants who are obese (BMI ≥ 30 kg/m^2^), there was an increasing proportion of people from body figure 1 to body figure 9, corresponding to those whose self-perceived body figure is greater than their desired body figure. On the contrary, the reverse is true for those adults whose perceived body figure rating is less than the desired one. Furthermore, in terms of sex differences, in overall assessment accuracy across the three BMI categories, there was no significant difference (*p* = 0.328), with 43.4% of men and 42.7% of women correctly identifying their BMI category. However, for the overweight, the corresponding percentages were 32.5 and 50.1%, and for normal BMI 91.9% and 85.1%, which are significantly different, with *p* < 0.001 in both cases. The proportion of satisfied men with perceived BMI equal to desired BMI was 61.1, 51.4, and 22.2% for BMI < 25 kg/m^2^, 25 ≤ BMI < 30 kg/m^2^, and BMI ≥ 30 kg/m^2^, respectively. The corresponding figures for women were 54.4, 30.7, and 12.6%.

It is clear that the proportion of people who wanted to reduce their body figure (*P* > Des) was higher for body figures 4–9 for the diagnosed compared to the non-diagnosed. This is true across all three BMI ranges, suggesting a more objective evaluation of body image and BMI from those who have been diagnosed. It also appeared that the previously diagnosed participants with a current self-perceived body figure in the overweight or obese range (FRS ≥ 5) were less satisfied (*P* = Des) with their current body figure than the non-diagnosed participants with a self-perceived body figure in the same FRS range. It can also be seen that the higher the correspondence between real and perceived BMI for obese/overweight people, the more likely that desired BMI was less than perceived or real BMI. For the obese/overweight whose difference between real and perceived BMI was large, their desired BMI coincides with their perceived BMI. This is clear in the highly significant (*p* < 0.001 using a chi-squared test) difference in satisfaction between the diagnosed and non-diagnosed participants of normal BMI, with the diagnosed being much less satisfied, independent of their current BMI state.

The proportion in each BMI category, where perceived and desired BMI are equal, is 57.7% for non-diagnosed normal, 41.8% for non-diagnosed overweight, and 18.1% for non-diagnosed obese. However, only 7.8% of obese people whose real and perceived BMI coincide have a desired BMI that is the same as their perceived one. On the other hand, 43.2% of individuals whose perceived BMI is normal have a desired BMI that is the same as their perceived one. Thus, those with a severe misperception of their BMI are almost six times more likely to be satisfied with their unhealthy state than those with a realistic view of it. In the case of the diagnosed, only 38.7% of diagnosed normal have a perceived BMI equal to their desired BMI with 19.2 and 8% being the corresponding percentages for the overweight and obese. Furthermore, there is a highly significant (*p* < 0.001 using a chi-squared test) difference in body figure satisfaction, as determined by taking the difference between desired body figure and perceived body figure, between the diagnosed and non-diagnosed with real BMI in the normal range, with the diagnosed being much less satisfied.

## Discussion

We observed the previously described discrepancy between real and perceived BMI (as proxied by the FRS and seen in Figure 1) among obese and overweight participants in the ENSANUT 2012 survey. If we take the standard assignment for the Stunkard scale of body figures 8 and 9 as corresponding to obesity (BMI ≥ 30 kg/m^2^), and 5–7 as overweight (25 ≤ BMI < 30 kg/m^2^), then only 8.8 and 6.1%, respectively, of the diagnosed and non-diagnosed with obesity correctly identified themselves as such, and only 55.5 and 41% of the overweight. This is obviously of great concern from a public health perspective, as has been pointed out by several authors (26, 27). On the basis that the percentage of participants that correctly identified their BMI category was considerably higher for the diagnosed versus the non-diagnosed, it has also been argued (28, 29) that a diagnosis of obesity/overweightness is a first step toward a more realistic appreciation of health state. This in turn can serve as a motivational basis for lifestyle change. However, when we calculated the percentage of non-diagnosed and diagnosed with BMI < 25 kg/m^2^ who correctly classify themselves as being in FRS categories 1–4, the percentages were 88.5 and 59.1%, respectively. Therefore, those diagnosed with BMI < 25 kg/m^2^ were even less accurate in their predictions than the non-diagnosed with BMI < 25 kg/m^2^. It has been argued on the basis of an analysis of the ENSANUT 2006 data (Easton, Stephens, and Román, 2017, submitted manuscript) that this may be attributed to the existence of two well-known cognitive biases—the self-serving bias, to explain the general underestimation of BMI among the obese, and the anchoring bias to explain the fact that the diagnosed with BMI < 25 kg/m^2^ perceive themselves to be of higher BMI than their non-diagnosed counterparts. Anchoring is a cognitive bias in which an initial piece of information is used as an anchor affecting all future decisions; in this case, a diagnosis by a medical professional causes adults who lose weight following a diagnosis to maintain a high self-perceived weight.

Turning now to the relation between perceived and desired BMI, Figure 2 shows that there was an ideal body figure rating, which varies only weakly as a function of real BMI independent of sex or diagnosis. Moreover, this average desired body figure rating, FRS categories 3 and 4, corresponds to the normal BMI category. In other words, on average, people were capable of determining and visualizing what corresponds to a normal BMI in terms of their desired BMI, but this contrasts strongly to their inability to determine normality in terms of their own perceived BMI. The conclusion is that people have a quite accurate BMI scale when it applies to the abstraction of a desired BMI for themselves, but a very different scale when it applies to their present perceived BMI. We hypothesize that this is due to the self-serving bias (30). In particular, in terms of desired BMI, the underweight body figures 1 and 2 were not favored on average. It is notable, however, that for all groups the desired body figure rating did increase with real BMI. We postulate that this may be related to a higher degree of realism as to the potential effort required to pass from their perceived BMI state to the desired one. This may also potentially explain why the desired figure rating for women is systematically slightly higher than that of men.

Given that a quite accurate notion of normality, in terms of desired body figure rating, exists among all BMI categories, it is reasonable to assume that the difference between perceived and desired body figure rating/BMI will serve as a necessary requirement for subsequent weight reduction efforts. In other words, for those whose perceived body figure rating is the same as their desired one, we may suppose that there will be no motivation to make any lifestyle and eating habit changes necessary to change their weight. In Table 2, we can clearly see the patterns associated with the relation between real, perceived, and desired BMI. For the BMI obese, we see that for those who perceived themselves as obese or overweight—body figure ratings 5–9—between 83 and 98% identified their desired BMI as being less than their real BMI. Thus, we hypothesize that for this group there will exist at least the basis for a justification that weight needs to be lost. However, 20.2% of non-diagnosed with obesity and 12.7% of diagnosed with obesity perceived their BMI in the body figure rating range 1–4, i.e., in the normal or underweight range. What is more alarming is that with respect to their desired BMI, the majority of these, 49.1% of non-diagnosed and 37% of diagnosed, were satisfied with their perceived body figure rating, or even wished to be identified with a higher body figure rating, associated with larger BMI!

If we take the difference between perceived and desired BMI as a potential motivator for change, then the degree of satisfaction with an extremely misperceived BMI is a potential barrier to such change. As shown in the results from Table 2, this is consistent with our point that, although a medical diagnosis leads the obese to a self-evaluation that is more consistent with their real BMI, this is better attributed to an anchoring effect (31) rather than to an improved ability in perceiving their BMI. This indicates that, as a population, the perception of normality for the diagnosed is actually less accurate overall than the non-diagnosed due to the contribution of those who lose weight and return to a normal BMI.

We would interpret the differences seen in Table 2 between sexes as a tendency for overweight men to identify themselves as normal when compared to women, and for normal women to identify themselves as overweight relative to men. For those whose perceived and desired BMI are equal, women are clearly much more dissatisfied across all BMI categories. This relatively larger dissatisfaction in women has been noted before (32, 33).

### Possible Intervention

In order to combat the effects discussed regarding cognitive biases, we believe it is important to create an intervention that can improve the accuracy of the self-perception of body figure, while, at the same time, helping create an awareness of significant weight gain before the onset of obesity. Due to the difficulty in losing weight once obesity has been reached (24), it is necessary to educate people regularly as to their current weight status. It has been shown in some studies (29, 34) that an intervention by a medical professional to make patients aware of their weight/body figure has a positive effect on motivating weight loss and helps to achieve accurate self-perception. Where a one off diagnosis can lead to overestimating current weight, should a diagnosed adult manage to return to a normal BMI, regular screening could help to correct erroneous self-perception and maintain awareness of current weight and weight gain to help stop the onset of obesity. It is also important to note that further research is required to design interventions such that an individual is made aware of significant weight gain and can therefore make a feasible intervention to revert the trend before reaching obesity.

### Limitations

We acknowledge as potential limitations of this research: (i) the data are cross-sectional, making causal inference more difficult, particularly in considering BMI differences across different groups not across a cohort of the same individuals; (ii) the male/female ratio for the ENSANUT respondents is skewed and thus not fully representative of the Mexican population; (iii) diagnosis of obesity by a health-care professional was self-reported and therefore subject to recall and other biases; and (iv) we assume that no other variable distinguishes the diagnosed from the non-diagnosed.

## Conclusion

Although the relation between perceived and real BMI has been extensively considered (17, 18, 25–28), the relation between these and desired BMI has been much less studied (19, 20), especially with large groups. In this paper, we have studied real, perceived, and desired BMI, as proxied by a Stunkard FRS, for a large sample of Mexican adults. We confirm that there is a general subestimation of weight among the obese/overweight, independent of sex or whether or not they have been diagnosed as obese/overweight by a health-care professional, which we partially attribute to the existence of the self-serving cognitive bias. However, the misperception of BMI is worse for men than women. It has been argued that diagnosis of obesity/overweight by a health-care professional can lead to a more realistic evaluation of BMI state. We have shown this not to be the case. Although the obese/overweight are more likely to self-identify as such, we believe that this is due to another cognitive bias—the anchoring bias—rather than an improved capacity to more accurately self-assess BMI. The evidence for this is that the diagnosed obese/overweight that managed to return to normal BMI were even worse than their non-diagnosed counterparts in assessing their real BMI. However, given that so few obese people manage to return to a normal state, from a public health perspective it may be that the anchoring effect of a diagnosis has a positive impact, in at least the self-perception of those most at risk is improved, even if the overall effect across all BMI categories is not. Additionally, periodic screenings, as opposed to a one off screening, could help to mitigate this anchoring effect.

We have shown that the degree of misperception of BMI is strongly correlated to whether or not an individual is satisfied with their perceived versus real BMI. The larger the deviation between perceived BMI and real BMI, the more likely it is that an individual is satisfied with their perceived BMI in that it represents their desired BMI. Indeed, there are a substantial proportion of cases where perceived BMI is so out of step with real BMI that the desired BMI is in the opposite direction to the logical one. For example, obese people who perceive themselves as thin but wish to be fatter. The relation between real, perceived, and desired BMI that we have studied shows the huge importance of self-perception, as it not only indicates a lack of self-awareness about a disease state—obesity—but relative to real BMI it also shows that that misperception correlates strongly with the degree of satisfaction that an individual has with his/her perceived BMI. If we take satisfaction as a proxy for potential inaction, then a natural consequence would be a lack of desire to address the health problem.

Our final conclusion is that it is important to devise widely available and simple screening methods by which people can be made aware of their real BMI, their perceived BMI, their desired BMI, and the differences between them. Furthermore, we believe that such screenings should be carried out periodically in order to avoid the negative effects associated with the anchoring bias. Such screenings could serve to identify those individuals whose perceptions and desires are especially far removed from reality.

## Author Contributions

All authors approve of the manuscript and its submission for publication, certifying that the work is original and their own. The contributions and work are as follows: JE and CS designed and conceived the investigation; JE and HS worked with the data and carried out statistical analysis; JE, CS, and HS participated in results interpretation and discussion; JE and CS wrote the manuscript; JE, CS, and HS revised the manuscript and approved the final version.

## Conflict of Interest Statement

The authors declare that the research was conducted in the absence of any commercial or financial relationships that could be construed as a potential conflict of interest.
